# Pregnancy and neonatal outcomes of women with disabilities: a nationwide population-based study in South Korea

**DOI:** 10.1038/s41598-020-66181-9

**Published:** 2020-06-08

**Authors:** Jae Eun Shin, Geum Joon Cho, Seongeun Bak, Sang Eun Won, Sung Won Han, Soo Bin Lee, Min-Jeong Oh, Sa Jin Kim

**Affiliations:** 1 Department of Obstetrics and Gynecology, College of Medicine, The Catholic University of Korea, Seoul, Korea; 2 Department of Obstetrics and Gynecology, College of Medicine, Korea University, Seoul, Korea; 3 School of Industrial Management Engineering, Korea University, Seoul, Korea

**Keywords:** Epidemiology, Outcomes research

## Abstract

We investigated (1) pregnancy and neonatal outcomes in women with and without disabilities, (2) time trends in deliveries, and (3) risks of pregnancy and neonatal complications among women with various disability types and severity. This was a nationwide population-based study merging the database of the Korea National Health Insurance claims, National Health Screening Program for Infants and Children, and Disability Registration System to compare perinatal outcomes in women with and without disabilities. Pregnancy and neonatal outcomes were analyzed during 2007 and 2015, as were time trends of deliveries. Multivariate logistic regression was used to evaluate risk of perinatal outcomes among women with various disability types and severities. Women with disabilities showed higher rates of cesarean section (aOR, 1.73; 95% CI, 1.69–1.77), hypertensive disorders (aOR, 1.74; 95% CI, 1.63–1.86), placenta abruption (aOR, 1.27; 95% CI, 1.12–1.45), placenta previa (aOR, 1.14; 95% CI, 1.05–1.24), stillbirths (aOR, 1.30; 95% CI, 1.17–1.45), preterm births (aOR, 1.67; 95% CI, 1.57–1.78), and LBW (aOR, 1.87; 95% CI, 1.78–1.97) than those without disabilities. From 2007 to 2015, although delivery rate in women with disabilities decreased steeply compared with that in women without disabilities, the rate of cesarean section increased in women with disabilities. Women with intellectual disability and those with vision impairment had the highest number of perinatal complications among women with various types of disabilities. Women with disability had more adverse pregnancy and neonatal outcomes than those without disabilities. Specific disability types & severities are more vulnerable to specific perinatal complications.

## Introduction

Worldwide, the prevalence of disabilities, defined as experiencing significant functional impairment in everyday life as an adult, is estimated to be 15.6%^1^. The global prevalence of disabilities among fertile women varies greatly from 6.4% to 12%, depending on the country and definition of disability^1–3^. In South Korea, data from the National Survey on Persons with Disabilities and Korean Statistical Information Service estimated that the prevalence of disabled persons was 2,668,411 (5.39%) nationwide^4^, and the proportion of disabled women among women of childbearing age was 1.39%^5^.

Emerging literature suggests that women with disabilities who become mothers are at an increased risk for poor maternal health, pregnancy complications, and adverse birth outcomes^6–8^. Some studies revealed that women with intellectual disabilities are associated with a higher incidence of cesarean rate^9^, preeclampsia^10^, and preterm births^9^. Women with hearing loss were more likely to have preterm labor and low birth weight (LBW)^11^, and visually impaired women had higher rates of cesarean deliveries than women without disabilities^12^. Furthermore, women with physical disabilities were confronted with medical and psychosocial risks, as well as barriers to prenatal care and parenting^13,14^. However, most of the studies have been performed with relatively small sample sizes, which are not representative of the general population, and used self-reported definitions of disability type and severity. These reasons make it difficult to draw conclusions to guide government policies and clinical practices. Moreover, no research to date has examined perinatal complications among women with specific disability type and severity.

In South Korea, universal health insurance is provided to almost everyone, and 94.1% of disabled persons are estimated to be registered to national registration systems according to the data from the latest National Survey on Persons with Disabilities conducted in 2017^4^. Diagnosis of disability and determination of disability type and severity are based on a specialist physician’s opinion, which empowered the clinical and accurate classification of the participants. In addition, data from many national organs can be linked to each other, so that information can be analyzed in various aspects.

In this study, we used nationally representative data to (1) compare the pregnancy and neonatal outcomes in women with and without disabilities, (2) assess time trends in deliveries and cesarean deliveries of women with and without disabilities, and (3) investigate the risks of pregnancy and neonatal complications among women with various types and differing levels of severity of the disabilities.

## Methods

### Characteristics of the data

This study was conducted by merging the databases of the Korea National Health Insurance (KNHI) claims, National Health Screening Program for Infants and Children (NHSP-IC) and Disability Registration System in Korea. The present study was performed in accordance to the latest version of the Declaration of Helsinki. The study was approved by the institutional review board of the Korea University (2019GR0136). Written informed consent was waived by the board as this is a register-based study on anonymized data where participants were neither identified nor contacted.

In Korea, 97% of the population is enrolled in the KNHI program, and the KNHI claims database contains all claims information for these individuals. Therefore, nearly all information on the diseases and treatments of participants can be obtained from this centralized database, with the exception of procedures that are not covered by insurance, such as cosmetic surgery.

The KNHI system also provides an NHSP-IC for all neonates for seven consecutive health examinations within defined age groups (4–9, 9–18, 18–30, 30–42, 42–54, 54–66, and 66–80 months). The NHSP-IC comprises two components: a health interview with the parents and a health examination of the offspring. Information on preterm birth and birthweight was obtained using the NHSP-IC health interview.

In 1988, the Korean government established the national Disability Registration System for individuals with disabilities, mainly for the provision of welfare benefits, based on their disability type and severity. Registration requires the submission of appropriate and validated documentation to a local National Pension Service office. The paperwork includes appraised results of the disability diagnosed by a specialist physician in the corresponding field, according to detailed criteria (there are 15 legally defined disability types and six severity levels), as defined by the national Disability Registration System. Level of severity for each disability is based on the limitation of functional status and graded from 1 (most severe) to 6 (least severe).

### Study population

**Figure 1:**
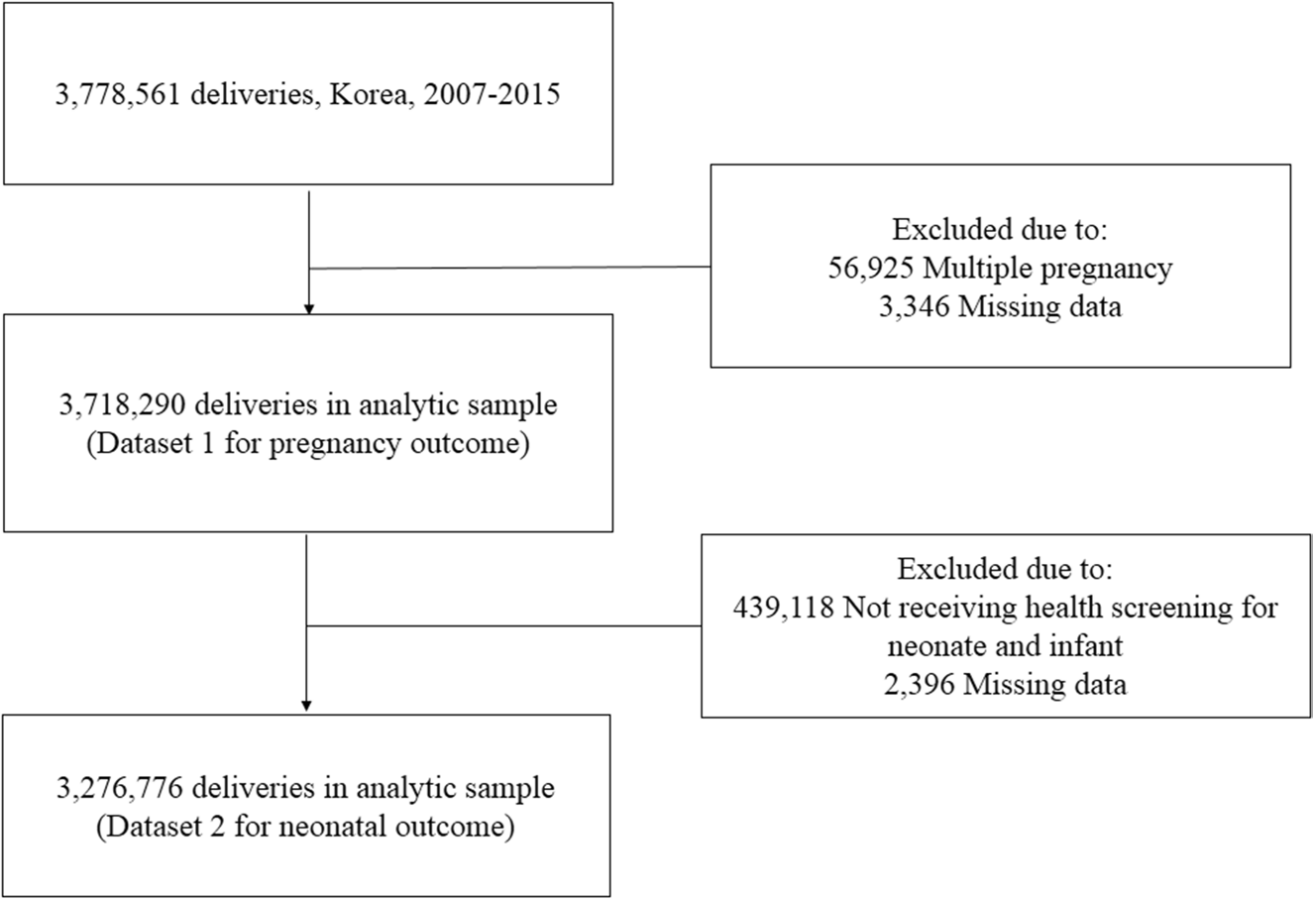
Flow diagram of the study.

Figure 1 shows the flowchart of study participants’ enrollment. Based on the KNHI claims dataset, we identified all women who delivered singletons (≥20 gestational weeks) between January 1, 2007, and December 31, 2015. To identify pregnancy outcomes, we excluded women who delivered multiple pregnancies or if data were missing (dataset 1). For neonatal outcomes, including preterm birth and birthweight, we merged the KNHI claims and NHSP-IC databases. Women were excluded from the analysis if their offspring did not undergo at least one of the seven consecutive NHSP-IC or if data were missing (dataset 2).

### Outcomes

Using data from the national Disability Registration System for individuals with disabilities, maternal disabilities were identified and categorized by severity, as mild (grades 4–6) and severe (grades 1–3), as well as by type.

Using dataset 1, pregnancy outcomes were identified. Available information included age, primiparity, delivery mode, hypertensive disorders, postpartum hemorrhage (PPH), placental abruption, placenta previa, and stillbirth based on International Classification of Diseases 10th Revision (ICD-10) codes. Hypertensive disorders in pregnancy was defined using the ICD codes for gestational hypertension, preeclampsia, superimposed preeclampsia, and eclampsia. Data regarding preterm birth and birthweight were extracted from dataset 2. Preterm birth was defined as gestational age <37 weeks, LBW was defined as birthweight <2.5 kg, and macrosomia was defined as birthweight >4.0 kg.

### Statistical analysis

Continuous and categorical variables are expressed as mean ± standard deviation and percentages, respectively. Clinical characteristics were compared using t-test for continuous variables and chi-square test for categorical variables. The significance level was defined as a p value less than 0.01. Multivariable logistic regression analysis was used to estimate the adjusted odds ratio (aOR) and 95% confidence intervals (CIs) to assess the correlation of maternal disability with pregnancy and neonatal outcomes. For multivariable analyses, a fixed set of known risk factors for perinatal outcome was adjusted for potential confounders. In the basic model, we first adjusted for maternal age and parity (model 1). In model 2, we adjusted for the variable in model 1 plus pre-gestational hypertension and pre-gestational diabetes. Statistical analyses were performed using SAS for Windows, version 9.4 (SAS Inc., Cary, NC, USA).

## Results

The total number of women with deliveries from 2007 to 2015 was 3,778,561. Among them, 0.72% of deliveries (26,697/3,718,290) involved women with disabilities. Characteristics of study participants are reported in Table 1, including a comparison between women with disabilities and those without disabilities. Women with disabilities tended to be older (31.65 ± 4.57 vs. 30.99 ± 3.91 years, p < 0.001), exhibit a lower percentage of primiparity (47.01 vs. 51.06%, p < 0.001), and a higher percentage of pre-gestational hypertension (8.45 vs. 3.29%, p < 0.001), and pre-gestational diabetes (9.75 vs. 4.90%, p < 0.001). They also included a higher percentage of women who had undergone cesarean sections (51.11 vs. 36.34%, p < 0.001), were diagnosed with hypertensive disorders (3.85 vs. 1.86%, p < 0.001), placental abruption (0.87 vs. 0.65%, p < 0.001), placenta previa (2.25 vs. 1.84%, p < 0.001) and stillbirths (1.27 vs. 0.91%, p < 0.001) compared to those without disabilities. Regarding neonatal outcomes, women with disabilities were associated with a higher percentage of preterm birth (4.57 vs. 2.58%, p < 0.001) and LBW (6.73 vs. 3.51%, p < 0.001) than those without disabilities.

**Table 1:** Baseline characteristics, and pregnancy and neonatal outcomes of the study population.

Dataset	Women without disabilities	Women with disabilities	p-value
Baseline characteristics	n = 3,691,593	n = 26,697	
Age, y	30.99 ± 3.91	31.65 ± 4.57	<0.001
Primiparity	1,884,850 (51.06)	12,549 (47.01)	<0.001
Pre-gestational hypertension	121,380 (3.29)	2,257 (8.45)	<0.001
Pre-gestational diabetes	180,874 (4.90)	2,604 (9.75)	<0.001
Pregnancy outcome	n = 3,691,593	n = 26,697	
Cesarean section	1,341,477 (36.34)	13,645 (51.11)	<0.001
Hypertensive disorders	68,668 (1.86)	1,027 (3.85)	<0.001
Postpartum hemorrhage	467,854 (12.67)	3,343 (12.52)	0.459
Placental abruption	23,964 (0.65)	231 (0.87)	<0.001
Placenta previa	67,766 (1.84)	602 (2.25)	<0.001
Stillbirth	33,508 (0.91)	339 (1.27)	<0.001
Neonatal outcome	n = 3,253,209	n = 23,567	
Preterm birth	84,081 (2.58)	1,077 (4.57)	<0.001
Male	1,676,208 (51.52)	12,209 (51.81)	0.39
Birth weight, kg	3.22 ± 0.46	3.13 ± 0.52	<0.001
Low birth weight	114,200 (3.51)	1,587 (6.73)	<0.001
Macrosomia	123,754 (3.80)	858 (3.64)	0.191

Values are expressed as mean ± standard deviation or as n (%).

**Figure 2:**
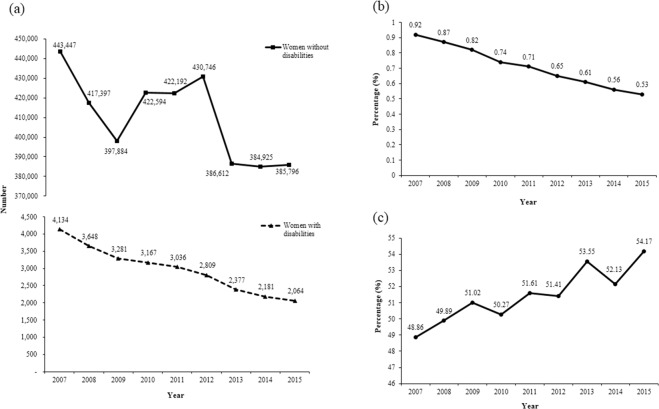
Trends in deliveries according to disability. (**a**) Total number of deliveries in women with disabilities and without disabilities. (**b**) Proportion of deliveries in women with disabilities to those in the study population. (**c**) Proportion of cesarean sections of women with disabilities compared to women without disabilities.

The number of total deliveries decreased from 447,581 in 2007 to 387,860 in 2015 (Fig. 2). The number of deliveries by women with disabilities declined by almost half (from 4,134 in 2007 to 2,064 in 2015), whereas a more modest decrease among women without disabilities (from 443,447 in 2007 to 385,796 in 2015) was observed. The proportion of deliveries in women with disabilities to the total deliveries in the entire study population decreased from 0.92% in 2007 to 0.53% in 2015. Among women with disabilities, the proportion of cesarean sections increased from 48.86% in 2007 to 54.17% in 2015.

Table 2 shows results of the logistic regression analysis for the relationship between disability status and perinatal outcome. After adjustment for age, parity, pre-gestational hypertension, and pre-gestational diabetes (model 2), women with disabilities showed higher rates of cesarean section (aOR, 1.73; 95% CI, 1.69–1.77), hypertensive disorders (aOR, 1.74; 95% CI, 1.63–1.86), placenta abruption (aOR, 1.27; 95% CI, 1.12–1.45), placenta previa (aOR, 1.14; 95% CI, 1.05–1.24), stillbirths (aOR, 1.30; 95% CI, 1.17–1.45), preterm births (aOR, 1.67; 95% CI, 1.57–1.78), and LBW (aOR, 1.87; 95% CI, 1.78–1.97) than those without disabilities. Women with severe disabilities had higher odds of cesarean section, hypertensive disorders, placenta abruption, stillbirth, preterm birth, and LBW than those with mild disabilities.

**Table 2:** Logistic regression analysis for disability status and severity predicting pregnancy outcomes and neonatal outcomes in South Korea (model 2).

	Pregnancy outcome						Neonatal outcome		
	Cesarean section	Hypertensive disorders	PPH	Placental abruption	Placenta previa	Stillbirth	Preterm birth	LBW	Macrosomia
	aOR (95% CI)	aOR (95% CI)	aOR (95% CI)	aOR (95% CI)	aOR (95% CI)	aOR (95% CI)	aOR (95% CI)	aOR (95% CI)	aOR (95% CI)
No disability	1	1	1	1	1	1	1	1	1
Any disability	1.73 (1.69, 1.77)	1.74 (1.63, 1.86)	0.99 (0.96, 1.03)	1.27 (1.12, 1.45)	1.14 (1.05, 1.24)	1.30 (1.17, 1.45)	1.67 (1.57, 1.78)	1.87 (1.78, 1.97)	0.92 (0.86, 1.01)
Severe disability (grades 1–3)	1.87 (180, 1.95)	2.31 (2.11, 2.53)	1.03 (0.97, 1.09)	1.49 (1.22, 1.81)	1.04 (0.90, 1.20)	1.41 (1.19, 1.67)	1.95 (1.77, 2.14)	2.40 (2.22, 2.59)	0.87 (0.77, 1.00)
Mild disability (grades 4–6)	1.65 (1.60, 1.70)	1.40 (1.29, 1.54)	0.97 (0.93, 1.02)	1.15 (0.96, 1.36)	1.20 (1.08, 1.32)	1.23 (1.07, 1.42)	1.52 (1.40, 1.64)	1.58 (1.47, 1.69)	0.95 (0.87, 1.04)

PPH, postpartum hemorrhage; LBW, low birth weight; aOR, adjusted odds ratio; CI, confidence interval.

Model 2: adjust for age, parity, pre-gestational hypertension and pre-gestational diabetes.

The basic model and model 1 (adjusted for age and parity) are described in supplementary Table S1.

Odds ratio (model 2) of pregnancy and neonatal outcomes according to disability types are demonstrated in Table 3. Patterns of perinatal outcomes varied with disability type. Higher rates of cesarean sections were noted in women with most types of disabilities, except for those with autism, liver disease, and facial disfiguration. When we compared the odds ratio of hypertensive disorders by disability type, the highest risk was seen in women with kidney disease (aOR, 7.42; 95% CI, 6.27–8.78) and modest risks in those with physical disabilities (aOR, 1.15; 95% CI, 1.03–1.29), brain lesions (aOR, 2.66; 95% CI, 2.12–3.34), visual impairment (aOR, 1.55; 95% CI, 1.31–1.85), intellectual disability (aOR, 2.71; 95% CI, 2.26–3.26), and mental disorder (aOR, 1.83; 95% CI, 1.35–2.50). In addition, women with physical (aOR, 1.22; 95% CI, 1.04–1.43) and intellectual (aOR, 2.64; 95% CI, 2.05–3.40) disabilities were associated with higher rates of stillbirths. Among neonatal outcomes, higher rates of LBW were noted in women with most types of disabilities, except those with hearing impairment, speech and language problems, autism, facial disfigurement, and ostomy. Women with internal organ problems (i.e., kidney disease, heart problems, respiratory problems, or liver disease) had a markedly higher rate of preterm births and LBW than those without disabilities, whereas women with external organ problems (physical, visual, brain lesion) or psychological (intellectual) disorders had slightly higher rates of preterm births and LBW than those without. Furthermore, among women with various types of disabilities, women with intellectual disabilities had the greatest number of perinatal complications, followed by those with vision impairment. Women with facial disfiguration and autism were not associated with any risk of complications.

**Table 3:** Logistic regression analysis for disability types predicting pregnancy and neonatal outcomes in South Korea (model 2).

	Pregnancy outcome						Neonatal outcome		
	Cesarean section	Hypertensive disorders	PPH	Placental abruption	Placenta previa	Stillbirth	Preterm birth	LBW	Macrosomia
	aOR (95% CI)	aOR (95% CI)	aOR (95% CI)	aOR (95% CI)	aOR (95% CI)	aOR (95% CI)	aOR (95% CI)	aOR (95% CI)	aOR (95% CI)
Physical impairment	2.05 (1.97, 2.12)	1.15 (1.03, 1.29)	0.98 (0.93, 1.04)	1.03 (0.84, 1.27)	1.14 (0.99, 1.25)	1.22 (1.04, 1.43)	1.40 (1.27, 1.54)	1.57 (1.45, 1.70)	0.91 (0.83, 1.01)
Brain lesions	1.64 (1.47, 1.82)	2.66 (2.12, 3.34)	0.95 (0.80, 1.12)	1.53 (0.90, 2.59)	0.89 (0.59, 1.34)	1.07 (0.63, 1.82)	1.68 (1.28, 2.19)	1.79 (1.43, 1.69)	0.85 (0.62, 1.17)
Visual impairment	1.391 (1.30, 1.47)	1.55 (1.31, 1.85)	1.03 (0.94, 1.12)	1.50 (1.12, 2.02)	1.35 (1.12, 1.63)	1.23 (0.94, 1.62)	1.42 (1.21, 1.68)	1.47 (1.28, 1.69)	1.04 (0.89, 1.22)
Hearing impairment	1.10 (1.02, 1.18)	1.11 (0.87, 1.43)	1.15 (1.04, 1.27)	1.26 (0.85, 1.86)	1.17 (0.92, 1.49)	1.16 (0.83, 1.64)	1.06 (0.83, 1.34)	1.09 (0.89, 1.33)	0.99 (0.81, 1.20)
Speech and language problems	1.77 (1.38, 2.05)	1.48 (0.72, 3.01)	1.05 (0.73, 1.50)	1.15 (0.29, 4.64)	2.21 (1.20, 4.04)	<0.01 (<0.01, >999.99)	1.64 (0.87, 3.09)	1.46 (0.82, 2.62)	1.00 (0.51, 1.95)
Intellectual disability	1.90 (1.76, 2.05)	2.71 (2.26, 3.26)	1.12 (1.01, 1.25)	1.65 (1.13, 2.39)	0.90 (0.66, 1.23)	2.64 (2.05, 3.40)	1.45 (1.16, 1.82)	2.63 (2.26, 3.06)	0.85 (0.67, 1.07)
Autism	2.32 (0.50, 10.67)	<0.01 (<0.01, >999.99)	0.01 (<0.01, >999.99)	<0.01 (<0.01, >999.99)	0.02 (<0.01, >999.99)	<0.01 (<0.01, >999.99)	0.02 (<0.01, >999.99)	0.01 (<0.01, >999.99)	0.02 (<0.01, >999.99)
Mental disorder	1.53 (1.34, 1.75)	1.83 (1.35, 2.50)	0.86 (0.70, 1.05)	1.21 (0.60, 2.42)	0.84 (0.53, 1.34)	0.41 (0.15, 1.08)	1.07 (0.72, 1.58)	1.44 (1.07, 1.95)	1.18 (0.86, 1.63)
Kidney disease	1.94 (1.67, 2.25)	7.42 (6.27, 8.78)	0.57 (0.43, 0.74)	2.25 (1.35, 3.76)	1.15 (0.73, 1.81)	1.25 (0.68, 2.14)	10.31 (8.71, 12.19)	11.28 (9.62, 13.24)	0.20 (0.09, 0.44)
Heart disease	1.93 (1.24, 3.00)	0.53 (0.13, 2.20)	0.74 (0.36, 1.53)	1.55 (0.22, 11.14)	1.26 (0.31, 5.13)	2.25 (0.55, 9.17)	5.66 (3.02, 10.60)	3.83 (2.01, 7.33)	1.41 (0.51, 3.86)
Respiratory disease	1.74 (1.14, 2.68)	<0.01 (<0.01, >999.99)	0.25 (0.08, 0.80)	<0.01 (<0.01, >999.99)	0.50 (0.07, 3.57)	<0.01 (<0.01, >999.99)	4.07 (2.02, 8.20)	4.70 (2.58, 8.56)	0.62 (0.15, 2.54)
Liver disease	1.27 (0.83, 1.93)	1.34 (0.48, 3.75)	0.68 (0.33, 1.40)	1.52 (0.21, 10.91)	3.46 (1.51, 3.57)	2.11 (0.52, 8.57)	3.75 (1.87, 7.54)	4.77 (2.67, 8.52)	1.51 (0.61, 3.73)
Facial disfigurement	1.33 (0.91, 1.95)	0.81 (0.20, 3.29)	0.49 (0.23, 1.06)	<0.01 (<0.01, >999.99)	1.29 (0.41, 4.07)	0.91 (0.13, 6.54)	0.72 (0.18, 2.93)	0.53 (0.13, 2.15)	1.60 (0.70, 3.65)
Ostomy	1.61 (1.04, 2.48)	1.01 (0.25, 4.18)	0.73 (0.35, 1.52)	3.53 (0.87, 14.38)	1.81 (0.57, 5.75)	<0.01 (<0.01, >999.99)	0.92 (0.23, 3.76)	2.19 (0.95, 5.05)	1.34 (0.49, 3.66)
Epilepsy	2.30 (1.86, 2.85)	1.14 (0.60, 2.14)	0.64 (0.44, 0.92)	1.18 (0.38, 3.68)	1.25 (0.64, 2.42)	1.91 (0.90, 4.03)	1.38 (0.79, 2.41)	1.56 (0.98, 2.48)	0.49 (0.29, 1.20)

PPH, postpartum hemorrhage; LBW, low birth weight; aOR, adjusted odds ratio; CI, confidence interval.

Model 2: adjust for age, parity, pre-gestational hypertension and pre-gestational diabetes.

The basic model and model 1 (adjusted for age and parity) are described in supplementary Table S1.

## Discussion

Our study confirms that women with disabilities have higher adverse perinatal outcomes than women without disabilities, and further showed that the pattern of perinatal complications differed according to disability severity and type. In addition, although delivery rates in women with disabilities decreased steeply during the study period, the rate of cesarean section increased.

Except data from small sample size studies, there are only a few population-based representative studies regarding pregnant women with overall disabilities. One study argued no difference in the risk of live births compared to non-disabled women^6^, whereas another study showed the increased risk of preterm births and LBW but not the risk of cesarean deliveries in women with disabilities^7^. Both these studies have strength in that they had a large sample size to prove the relationship, however they had limitations with regards to definition of disability, as they were based on self-reported interviews, which are subjective and unclear.

This study showed that women with intellectual disabilities had the greatest number of complications among women with disabilities. In most previous studies, pregnancies involving women with intellectual disabilities are associated with adverse perinatal outcomes, such as high rates of cesarean delivery, preterm delivery, LBW, hypertensive disorders of pregnancy, and stillbirths^9,10,15–18^. In comparison, one study performed in England argued that the outcomes of neonates were generally reassuring^19^. The latter study was limited, in that it had a low prevalence of women with disabilities (0.09%) compared to other studies, which might have resulted in misleading conclusions. Although it was unclear why the most adverse perinatal outcomes were observed in women with an intellectual disability compared to women with various types of disabilities, we assume that cognitive limitation due to intellectual disability interferes with adequate prenatal care, antenatal care, and medical service. Although all pregnant women should have access to a primary obstetric care provider to prevent or minimize perinatal adverse outcomes^20^, women with intellectual disabilities have limited or delayed medical services because they cannot recognize the signs and symptoms of pregnancy in itself or pregnancy complications. In other studies, discussing disparities in health care access of preventive services by disability type, women with cognitive disabilities were least likely to utilize reproductive medical care (Pap test and mammogram)^21–23^.

Women with vision impairment had the second most common perinatal complications. To date, only two studies reported on women with visual impairment in relation to perinatal outcome, and they showed inconsistent results. Darney *et al*.^24^ showed similar results to our study in that women with visual disabilities had adverse pregnancy outcomes (i.e., the highest proportions of pregestational and gestational diabetes, hypertension, and gestational hypertension, preterm births, and cesarean delivery). In Ofir’s study^12^, women with visual disabilities were associated with higher rates of severe preeclampsia, gestational diabetes, preterm delivery, cesarean delivery, congenital malformation, and very LBW (<1500 g) in a univariate study; however, they had only two independent risk factors (cesarean delivery and transfusion) after controlling for all confounders. A possible explanation for this is women with visual impairments might already have underlying or autoimmune diseases, which could affect prognosis of the pregnancy. Ofir *et al*.^12^ also noted that a high proportion of primary cesarean deliveries might have been related to diabetes. Unfortunately, we could not explore underlying diseases directly in our analysis, as these data did not address the reason of diagnosis or attributable barriers. Therefore, further prospective studies of controlling underlying conditions are needed to better clarify the relationship between visually impaired women and perinatal outcomes.

Another important finding from our study is that cesarean delivery was the most common complication in pregnant women in all disability types in South Korea, and women with kidney disease had the highest odds of cesarean delivery. In addition, almost half of the women with disabilities delivered by cesarean section, and the rate increased. Our finding is consistent with that of previous studies reporting that women with disabilities are more likely to have a cesarean delivery than those without disabilities. In previous studies, women with physical disabilities had higher risk of cesarean delivery than those without disabilities^14^, and women with intellectual disabilities showed an increased risk of cesarean delivery compared to those without disabilities^9,15,17^. A study that compared the risk of cesarean delivery among women with various disability groups showed that women with physical disabilities had the highest odds of cesarean delivery among the subgroups^24^, unlike our study in which women with kidney disease had the highest odds. Previous studies included only four disability types (physical, vision, hearing, and intellectual disabilities); however, our study included various types of internal and external organs and psychologic disabilities. The results demonstrate that the risk of cesarean delivery is high in almost all disability types and almost half of women with disabilities have a cesarean delivery; therefore, we suspect that the decision of delivery method is not due to obstetric indication, rather due to other factors such as possibly being imposed by the healthcare providers or patients or their families. Previous studies also noted that other factors, including maternal difficulties with communication and comprehension and lack of medical information and understanding, might influence the self-determination of medical treatment in women with disabilities^14,25–27^.

Although women with disabilities are bearing children at increasing rates in other countries^3,28^, the pregnancy rate of women with disabilities in South Korea is decreasing. The overall pregnancy rate is decreasing in Korea; however, the decreasing rate is more rapid among women with disabilities than those without, thereby widening the disparity between the two groups. Although disability-related policies and services to improve accessibility to medical services in women with disabilities have been expanded in South Korea (financial support and welfare services)^29,30^, 50% of survey respondents answered that the government’s support was unsatisfactory and that the most needed service for women with disabilities was a pregnancy-related service^4^. In addition, another plausible explanation of disparity is the stigma and negative reactions from family members and the society with respect to disabled pregnant women. Women with disabilities frequently experience negative reactions to the idea of becoming parents from family members, health care providers, and the society^31^. A total of 34% of the population with disabilities needed help from others and that help was mostly obtained from family members (81.9%) in the survey^4^. Therefore, willingness of becoming pregnant can be abandoned by women with disabilities themselves or rejected by family members, because it can be a burden to the family. The government policy should be developed to lessen the burden of family help to women with disabilities.

The study has several limitations. First, there is the possibility of erroneous data in NHID; however, we could not prove the accuracy of the coded diagnosis or outcomes. Second, our study has a limitation common to other observational studies using administrative data. We were limited to the information in the registry-based datasets, which was comprised of limited data of coded diagnosis and outcomes. The relationship between maternal disability and perinatal outcomes could be impacted by variables not available or recorded in our dataset. No data on prior pregnancies or previous perinatal complications were available. Detailed information about maternal and fetal morbidity and mortality during pregnancy could not be identified either. Third, information about demographic factors and socioeconomic and lifestyle variables that may influence perinatal outcomes were not available from NHID. Furthermore, our study population may have multiple disabilities, but provided data are limited. Finally, the study population for the analysis of pregnancy and neonatal outcomes was different, as we removed some women via our exclusion criteria. However, it is unlikely that this would impact our conclusion, as the number of excluded patients was relatively small, compared to the total study population.

Our findings suggest that specific disability types & severities are more vulnerable to specific perinatal complications. This conclusion may influence the future direction of government policies and medical guidelines, potentially having a worldwide effect. Additional research is needed to identify specific risk factors for adverse perinatal outcomes in the subpopulation of disabled women after controlling for various socioeconomic factors.

## Supplementary information

Dataset 1.

## Data Availability

The data are available on request from the corresponding author.
