# Supplementary material for: Pregnancy and neonatal outcomes of women with disabilities: a nationwide population-based study in South Korea
Source: Sci Rep. 2020 Jun 8;10:9200. doi: 10.1038/s41598-020-66181-9 (PMC7280207; doi:10.1038/s41598-020-66181-9)
Supplement: Supplementary file 1 — Dataset 1. [file 41598_2020_66181_MOESM1_ESM.docx]

**Pregnancy and neonatal outcomes of women with disabilities: a nationwide population-based study in South Korea**

Jae Eun Shin^1^, Geum Joon Cho^2^, Seongeun Bak^1^, Sang Eun Won^1^, Sung Won Han^3^, Soo Bin Lee^3^, Min-Jeong Oh^2^, Sa Jin Kim^1^

**Table S1** Logistic regression analysis for disability severity and types for predicting pregnancy outcomes and neonatal outcomes in South Korea (basic model and model 1)

|  | | Pregnancy outcome | | | | | | | | | | | | | | | | | | | | | | |  | | | | | | | | | | | | | | | | | | | | | | | | | | Neonatal outcomes | | | | | | | | | | | | | | | | | | | | | | | | | | |
| --- | --- | --- | --- | --- | --- | --- | --- | --- | --- | --- | --- | --- | --- | --- | --- | --- | --- | --- | --- | --- | --- | --- | --- | --- | --- | --- | --- | --- | --- | --- | --- | --- | --- | --- | --- | --- | --- | --- | --- | --- | --- | --- | --- | --- | --- | --- | --- | --- | --- | --- | --- | --- | --- | --- | --- | --- | --- | --- | --- | --- | --- | --- | --- | --- | --- | --- | --- | --- | --- | --- | --- | --- | --- | --- | --- | --- | --- |
|  |  | Cesarean section | | | | | | | | | Preeclampsia | | | | | | | PPH | | | | | | | Placental abruption | | | | | | | | Placenta previa | | | | | | | | | Stillbirth | | | | | | | | | Preterm birth | | | | | | | | | LBW | | | | | | | | | LGA | | | | | | | | |
|  |  | N (%) | | | Crude OR  (95% CI) | | | aOR  (95% CI) | | | N (%) | Crude OR (95% CI) | | | aOR  (95% CI) | | | N (%) | Crude OR (95% CI) | | | aOR  (95% CI) | | | N (%) | | Crude OR (95% CI) | | | aOR  (95% CI) | | | N (%) | | | Crude OR (95% CI) | | | aOR  (95% CI) | | | N (%) | | | Crude OR (95% CI) | | | aOR  (95% CI) | | | N (%) | | | Crude OR (95% CI) | | | aOR  (95% CI) | | | N (%) | | | Crude OR (95% CI) | | | aOR  (95% CI) | | | N (%) | | | Crude OR (95% CI) | | | aOR  (95% CI) | | |
|  |  |  |  |  |  |  |  | model 1 | | |  |  |  |  | model 1 | | |  |  |  |  | model 1 | | |  |  |  |  |  | model 1 | | |  |  |  |  |  |  | model 1 | | |  |  |  |  |  |  | model 1 | | |  |  |  |  |  |  | model 1 | | |  |  |  |  |  |  | model 1 | | |  |  |  |  |  |  | model 1 | | |
| **By disability severity** | | | | | | | | | | | | | | | | | | | | | | | | | | | | | | | | | | | | | | | | | | | | | | | | | | | | | | | | | | | | | | | | | | | | | | | | | | | | | |
| No disability | 1,341,477 (36.34) | | | 1 | | | 1 | | | | 68668  (1.86) | | 1 | | | 1 | | 467,854 (12.67) | | 1 | | | 1 | | 23,964 (0.65) | | | 1 | | | 1 | | | 67,766 (1.84) | | | 1 | | | 1 | | | 33,508 (0.91) | | | 1 | | | 1 | | | 84,081  (2.58) | | | 1 | | | 1 | | | 114,200 (3.51) | | | 1 | | | 1 | | | 123,754 (38.80) | | | 1 | | | 1 | |
| Any disability |  | | | 1.83  (1.79, 1.88) | | | 1.78  (1.73, 1.82) | | | |  | | 2.11  (1.98, 2.25) | | | 2.06  (1.93, 2.19) | |  | | 0.99  (0.95, 1.02) | | | 0.99  (0.96, 1.03) | |  | | | 1.34  (1.17, 1.52) | | | 1.31  (1.15, 1.50) | | |  | | | 1.23  (1.14, 1.34) | | | 1.15  (1.06, 1.25) | | |  | | | 1.41  (1.26, 1.57) | | | 1.35  (1.22, 1.51) | | |  | | | 1.81  (1.70, 1.92) | | | 1.77  (1.67, 1.88) | | |  | | | 1.99  (1.89, 2.09) | | | 1.96  (1.86, 2.06) | | |  | | | 0.96  (0.89, 1.02) | | | 0.94  (0.88, 1.01) | |
| Severe disability  (grades 1-3) | 5,200 (52.42) | | | 1.93  (1.86, 2.01) | | | 1.93  (1.86, 2.01) | | | | 509 (5.13) | | 2.85  (2.61, 3.12) | | | 2.81  (2.57, 3.07) | | 1,285 (12.95) | | 1.03  (0.97, 1.09) | | | 1.03  (0.97, 1.09) | | 100 (1.01) | | | 1.56  (1.28, 1.90) | | | 1.55  (1.27, 1.88) | | | 201 (2.03) | | | 1.11  (0.96, 1.27) | | | 1.05  (0.91, 1.21) | | | 136 (1.37) | | | 1.52  (1.28, 1.80) | | | 1.49  (1.26, 1.76) | | | 453 (5.30) | | | 2.11  (1.92, 2.32) | | | 2.09  (1.90, 2.30) | | | 724 (8.48) | | | 2.11  (1.92, 2.32) | | | 2.53  (2.34, 2.73) | | | 293 (3.43) | | | 0.90  (0.80, 1.01) | | | 0.89  (0.79, 1.00) | |
| Mild disability (grades 4-6) | 8,445 (50.33) | | | 1.78  (1.72, 1.83) | | | 1.69  (1.64, 1.74) | | | | 518 (3.09) | | 1.68  (1.54, 1.84) | | | 1.63  (1.49, 1.78) | | 2,058 (12.27) | | 0.96  (0.92, 1.01) | | | 0.97  (0.93, 1.02) | | 131 (0.78) | | | 1.20  (1.01, 1.43) | | | 1.18  (0.99, 1.40) | | | 401 (2.39) | | | 1.31  (1.19, 1.45) | | | 1.21  (1.09, 1.33) | | | 203 (1.21) | | | 1.34  (1.16, 1.54) | | | 1.28  (1.11, 1.47) | | | 624 (4.15) | | | 1.63  (1.51, 1.77) | | | 1.59  (1.47, 1.73) | | | 863 (5.74) | | | 1.63  (1.51, 1.77) | | | 1.64  (1.53, 1.76) | | | 565 (3.76) | | | 0.99  (0.91, 1.08) | | | 0.97  (0.89, 1.05) | |
| **By disability type** | | | | | | | | | | | | | | | | | | | | | | | | | | | | | | | | | | | | | | | | | | | | | | | | | | | | | | | | | | | | | | | | | | | | | | | | | | | | | |
| Physical impairment | | | 7,003 (55.90) | | | 2.22  (2.14, 2.30) | | | 2.08  (2.01, 2.15) | 312 (2.49) | | | | 1.35 (1.20, 1.51) | | | 1.27  (1.14, 1.43) | 1,544 (12.33) | | | 0.97  (0.92, 1.02) | | | 0.98  (0.93, 1.04) | | 88 (0.70) | | | 1.08  (0.88, 1.34) | | | 1.05  (0.85, 1.30) | | | 286 (2.28) | | | 1.25  (1.11, 1.41) | | | 1.12  (0.10, 1.26) | | | 150 (1.20) | | | 1.32  (1.13, 1.56) | | | 1.25  (1.06, 1.47) | | | 429 (3.83) | | | 1.50  (1.36, 1.65) | | | 1.45  (1.31, 1.60) | | | 640 (5.71) | | | 1.50  (1.36, 1.65) | | | 1.61  (1.49, 1.75) | | | 407 (3.63) | | | 0.95  (0.86, 1.05) | | | 0.93  (0.84, 1.02) |
| Brain lesions | | | 661 (50.27) | | | 1.77  (1.59, 1.97) | | | 1.72  (1.54, 1.92) | 84 (6.39) | | | | 3.60 (2.89, 4.49) | | | 3.46  (2.77, 4.33) | 158 (12.02) | | | 0.94  (0.80, 1.11) | | | 0.95  (0.80, 1.12) | | 14 (1.06) | | | 1.65  (0.97, 2.79) | | | 1.63  (0.96, 2.75) | | | 23 (1.75) | | | 0.95  (0.63, 1.44) | | | 0.90  (0.59, 1.36) | | | 14 (1.06) | | | 1.18  (0.69, 1.99) | | | 1.15  (0.68, 1.95) | | | 56 (4.78) | | | 1.89  (1.45, 2.47) | | | 1.86  (1.42, 2.43) | | | 79 (6.74) | | | 1.89  (1.45, 2.47) | | | 1.95  (1.55, 2.45) | | | 39 (3.33) | | | 0.87  (0.63, 1.20) | | | 0.86  (0.63, 1.19) |
| Visual impairment | | | 1984  (45.28) | | | 1.45  (1.37, 1.54) | | | 1.41  (1.33, 1.50) | 138 (3.15) | | | | 1.72 (1.45, 2.03) | | | 1.71  (1.45, 2.03) | 567 (12.94) | | | 1.02  (0.94, 1.12) | | | 1.03  (0.94, 1.12) | | 44 (1.00) | | | 1.55  (1.15, 2.09) | | | 1.53  (1.14, 2.06) | | | 114 (2.60) | | | 1.43  (1.19, 1.72) | | | 1.36  (1.13, 1.64) | | | 52 (1.19) | | | 1.31  (1.00, 1.72) | | | 1.27  (0.96, 1.67) | | | 149 (3.80) | | | 1.49  (1.26, 1.75) | | | 1.47  (1.25, 1.74) | | | 205 (5.23) | | | 1.49  (1.26, 1.75) | | | 1.51  (1.31, 1.74) | | | 159 (4.06) | | | 1.07  (0.91, 1.25) | | | 1.05  (0.90, 1.23) |
| Hearing impairment | | | 1,179 (39.03) | | | 1.12  (1.04, 1.21) | | | 1.11  (1.03, 1.19) | 64 (2.12) | | | | 1.14 (0.89, 1.46) | | | 1.16  (0.90, 1.49) | 433 (14.33) | | | 1.15  (1.04, 1.28) | | | 1.15  (1.04, 1.27) | | 25 (0.83) | | | 1.28  (0.86, 1.89) | | | 1.26  (0.85, 1.87) | | | 67 (2.22) | | | 1.21  (0.95, 1.55) | | | 1.17  (0.92, 1.49) | | | 33 (1.09) | | | 1.21  (0.86, 1.70) | | | 1.17  (0.83, 1.65) | | | 73 (2.78) | | | 1.08  (0.85, 1.36) | | | 1.07  (0.85, 1.35) | | | 101 (3.84) | | | 1.08  (0.85, 1.36) | | | 1.10 (0.90, 1.34) | | | 100 (3.80) | | | 1.00  (0.82, 1.22) | | | 0.99  (0.81, 1.21) |
| Speech and language problems | | | 134  (51.54) | | | 1.86  (1.46, 2.38) | | | 1.79  (1.40, 2.29) | 8 (3.08) | | | | 1.68 (0.83, 3.39) | | | 1.60  (0.79, 3.23) | 34 (13.08) | | | 1.04  (0.72, 1.49) | | | 1.05  (0.73, 1.50) | | 2 (0.77) | | | 1.19  (0.30, 4.77) | | | 1.17  (0.29, 4.69) | | | 11 (4.23) | | | 2.36  (1.29, 4.32) | | | 2.21  (1.21, 4.06) | | | 0 (0) | | | <0.01 (<0.01, >999.99) | | | <0.01 (<0.01, >999.99) | | | 10 (4.39) | | | 1.73  (0.92, 3.26) | | | 1.69  (0.90, 3.18) | | | 12 (5.26) | | | 1.73  (0.92, 3.26) | | | 1.49  (0.83, 2.67) | | | 9 (3.95) | | | 1.04  (0.53, 2.02) | | | 1.02  (0.53, 1.99) |
| Intellectual disability | | | 1,296 (48.41) | | | 1.64  (1.52, 1.77) | | | 1.94  (1.79, 2.09) | 126 (4.71) | | | | 2.61 (2.18, 3.12) | | | 2.98  (2.49, 3.57) | 386 (14.42) | | | 1.16  (1.04, 1.29) | | | 1.12  (1.01, 1.25) | | 28 (1.05) | | | 1.62  (1.12, 2.35) | | | 1.68  (1.16, 2.44) | | | 40 (1.49) | | | 0.81  (0.59, 1.11) | | | 0.91  (0.66, 1.24) | | | 62 (2.32) | | | 2.59  (2.01, 3.33) | | | 2.72  (2.12, 3.50) | | | 79 (3.60) | | | 1.41  (1.12, 1.76) | | | 1.50  (1.20, 1.88) | | | 183 (8.33) | | | 1.41  (1.12, 1.76) | | | 2.69  (2.32, 3.14) | | | 70 (3.19) | | | 0.83  (0.66, 1.06) | | | 0.86  (0.68, 1.09) |
| Autism | | | 4 (57.14) | | | 2.34  (0.52, 10.43) | | | 2.34  (0.50, 10.89) | 0 (0) | | | | <0.01 (<0.01, >999.99) | | | <0.01 (<0.01, >999.99) | 0 | | | <0.01 (<0.01, >999.99) | | | 0.01 (<0.01, >999.99) | | 0 (0) | | | <0.01 (<0.01, >999.99) | | | <0.01 (<0.01, >999.99) | | | 0 (0) | | | 0.02  (<.01, >999.99) | | | 0.02  (<0.01, >999.99) | | | 0 (0) | | | <0.01 (<0.01, >999.99) | | | <0.01 (<0.01, >999.99) | | | 0 (0) | | | 0.02  (<.01, >999.99) | | | 0.02  (<0.01, >999.99) | | | 0 (0) | | | 0.02  (<.01, >999.99) | | | 0.02  (<0.01, >999.99) | | | 0 (0) | | | 0.02  (<.01, >999.99) | | | 0.02  (<0.01, >999.99) |
| Mental disorder | | | 479 (51.45) | | | 1.86  (1.63, 2.11) | | | 1.60  (1.40, 1.82) | 44 (4.73) | | | | 2.62 (1.93, 3.54) | | | 2.23  (1.65, 3.02) | 100 (10.74) | | | 0.83  (0.67, 1.02) | | | 0.86  (0.70, 1.05) | | 8 (0.86) | | | 1.33  (0.66, 2.66) | | | 1.26  (0.63, 2.53) | | | 18 (1.93) | | | 1.05  (0.66, 1.68) | | | 0.85  (0.53, 1.36) | | | 4 (0.43) | | | 0.47  (0.18, 1.26) | | | 0.43  (0.16, 1.15) | | | 26 (3.21) | | | 1.25  (0.85, 1.85) | | | 1.16  (0.79, 1.72) | | | 46 (5.69) | | | 1.25  (0.85, 1.85) | | | 1.54  (1.14, 2.07) | | | 39 (4.82) | | | 1.28  (0.93, 1.77) | | | 1.22  (0.89, 1.69) |
| Kidney disease | | | 462 (60.95) | | | 2.73  (2.36, 3.16) | | | 2.55  (2.20, 2.96) | 231 (30.47) | | | | 23.13 (19.81, 27.00) | | | 20.93  (17.88, 24.51) | 56 (7.39) | | | 0.55  (0.42, 0.72) | | | 0.57  (0.43, 0.74) | | 15 (1.98) | | | 3.09  (1.85, 5.15) | | | 3.03  (1.82, 5.05) | | | 19 (2.51) | | | 1.38  (0.87, 2.17) | | | 1.24  (0.78, 1.95) | | | 12 (1.58) | | | 1.76 (0.99, 3.11) | | | 1.73  (0.98, 3.06) | | | 208 (31.23) | | | 17.12  (14.53, 20.17) | | | 16.41  (13.92, 19.34) | | | 256 (38.44) | | | 17.12  (14.53, 20.17) | | | 16.37  (13.99, 19.15) | | | 6 (0.90) | | | 0.23  (0.10, 0.51) | | | 0.23  (0.10, 0.51) |
| Heart disease | | | 46 (55.42) | | | 2.18  (1.41, 3.36) | | | 2.24  (1.45, 3.48) | 2 (2.41) | | | | 1.30 (0.32, 5.30) | | | 1.25  (0.31, 5.09) | 8 (9.64) | | | 0.74  (0.36, 1.52) | | | 0.74  (0.36, 1.53) | | 1 (1.20) | | | 1.87  (0.26, 13.41) | | | 1.88  (0.26, 13.53) | | | 2 (2.41) | | | 1.32  (0.33, 5.37) | | | 1.31 (0.32, 5.36) | | | 2 (2.41) | | | 2.70  (0.66, 10.96) | | | 2.76  (0.68, 11.24) | | | 12 (16.44) | | | 7.42  (3.99, 13.77) | | | 7.40  (3.98, 13.75) | | | 11 (15.07) | | | 7.42  (3.99, 13.77) | | | 4.83  (2.54, 9.19) | | | 4 (5.48) | | | 1.47  (0.54, 4.02) | | | 1.48  (0.54, 4.06) |
| Respiratory disease | | | 49 (55.68) | | | 2.20  (1.45, 3.35) | | | 1.88  (1.23, 2.87) | 0 (0) | | | | <0.01 (<0.01, >999.99) | | | <0.01 (<0.01, >999.99) | 3 (3.41) | | | 0.24  (0.08, 0.77) | | | 0.25  (0.08, 0.80) | | 0 (0) | | | <0.01 (<0.01, >999.99) | | | <0.01 (<0.01, >999.99) | | | 1 (1.14) | | | 0.62  (0.09, 4.41) | | | 0.51  (0.07, 3.64) | | | 0 (0) | | | <0.01 (<0.01, >999.99) | | | <0.01 (<0.01, >999.99) | | | 9 (11.84) | | | 5.06  (2.53, 10.15) | | | 4.65  (2.32, 9.33) | | | 13 (17.11) | | | 5.06  (2.53, 10.15) | | | 5.20  (2.86, 9.47) | | | 2 (2.63) | | | 0.68  (0.17, 2.78) | | | 0.65  (0.16, 2.64) |
| Liver disease | | | 42 (46.15) | | | 1.50  (0.99, 2.27) | | | 1.41  (0.93, 2.13) | 4 (4.40) | | | | 2.43 (0.89, 6.61) | | | 2.20  (0.81, 6.02) | 8 (8.79) | | | 0.66  (0.32, 1.37) | | | 0.68  (0.33, 1.40) | | 1 (1.10) | | | 1.70  (0.24, 12.20) | | | 1.67  (0.23, 12.02) | | | 6 (6.59) | | | 3.78  (1.65, 8.65) | | | 3.55  (1.54, 8.13) | | | 2 (2.20) | | | 2.45  (0.60, 9.96) | | | 2.42  (0.60, 9.83) | | | 9 (10.98) | | | 4.65  (2.33, 9.29) | | | 4.49  (2.25, 8.99) | | | 14 (17.07) | | | 4.65  (2.33, 9.29) | | | 5.45  (3.06, 9.70) | | | 5 (6.10) | | | 1.64  (0.67, 4.06) | | | 1.62  (0.66, 4.01) |
| Facial disfigurement | | | 51 (47.22) | | | 1.57  (1.07, 2.29) | | | 1.36  (0.93, 2.00) | 2 (1.85) | | | | 1.00 (0.25, 4.03) | | | 0.89  (0.22, 3.61) | 7 (6.48) | | | 0.48  (0.22, 1.03) | | | 0.49  (0.23, 1.06) | | 0 (0) | | | <0.01 (<0.01, >999.99) | | | <0.01 (<0.01, >999.99) | | | 3 (2.78) | | | 1.53  (0.49, 4.81) | | | 1.30  (0.41, 4.09) | | | 1 (0.93) | | | 1.02  (0.14, 7.31) | | | 0.94  (0.13, 6.75) | | | 2 (2.08) | | | 0.80  (0.20, 3.25) | | | 0.76  (0.19, 3.07) | | | 2 (2.08) | | | 0.80  (0.20, 3.25) | | | 0.55  (0.14, 2.24) | | | 6 (6.25) | | | 1.69  (0.74, 3.85) | | | 1.62  (0.71, 3.70) |
| Ostomy | | | 42 (50.00) | | | 1.75  (1.14, 2.69) | | | 1.65  (1.07, 2.54) | 2 (2.38) | | | | 1.29 (0.32, 5.23) | | | 1.21  (0.30, 4.94) | 8 (9.52) | | | 0.73  (0.35, 1.50) | | | 0.73  (0.35, 1.52) | | 2 (2.38) | | | 3.73  (0.92, 15.18) | | | 3.65  (0.90, 14.83) | | | 3 (3.57) | | | 1.98  (0.63, 6.27) | | | 1.83  (0.58, 5.79) | | | 0 (0) | | | <0.01 (<0.01, >999.99) | | | <0.01 (<0.01, >999.99) | | | 2 (2.63) | | | 1.02  (0.25, 4.15) | | | 0.99  (0.24, 4.03) | | | 6 (7.89) | | | 1.02  (0.25, 4.15) | | | 2.30  (0.10, 5.30) | | | 4 (5.26) | | | 1.41  (0.51, 3.85) | | | 1.36  (0.50, 3.73) |
| Epilepsy | | | 213 (58.36) | | | 2.45  (1.99, 3.02) | | | 2.44  (1.98, 3.02) | 10 (2.74) | | | | 1.49 (0.79, 2.79) | | | 1.47  (0.78, 2.76) | 31 (8.49) | | | 0.64  (0.44, 0.92) | | | 0.64  (0.44, 0.92) | | 3 (0.82) | | | 1.27  (0.41, 3.95) | | | 1.25  (0.40, 3.89) | | | 9 (2.47) | | | 1.35  (0.70, 2.62) | | | 1.27  (0.65, 2.45) | | | 7 (1.92) | | | 2.14  (1.01, 4.51) | | | 2.06  (0.98, 4.35) | | | 13 (3.96) | | | 1.56  (0.89, 2.71) | | | 1.54  (0.89, 2.69) | | | 19 (5.79) | | | 1.56  (0.89, 2.71) | | | 1.69  (1.06, 2.69) | | | 8 (2.44) | | | 0.63  (0.31, 1.28) | | | 0.62  (0.31, 1.26) |

PPH, postpartum hemorrhage; LBW, low birth weight; LGA, large size for gestational age; aOR, adjusted odds ratio; CI, confidence interval.

Model 1: adjust for age and parity
